# Evolutionary conservation of the DRACH signatures of potential N6-methyladenosine (m^6^A) sites among influenza A viruses

**DOI:** 10.1038/s41598-021-84007-0

**Published:** 2021-02-25

**Authors:** Mahmoud Bayoumi, Muhammad Munir

**Affiliations:** Division of Biomedical and Life Sciences, Lancaster University, Lancaster, LA1 4YG UK

**Keywords:** Virology, Infectious diseases, Respiratory tract diseases

## Abstract

The addition of a methyl group to the N6-position of adenosine (m^6^A) is considered one of the most prevalent internal post-transcriptional modifications and is attributed to virus replication and cell biology. Viral epitranscriptome sequencing analysis has revealed that hemagglutinin (HA) mRNA of H1N1 carry eight m^6^A sites which are primarily enriched in 5′-DRACH-3′ sequence motif. Herein, a large-scale comparative m^6^A analysis was conducted to investigate the conservation patterns of the DRACH motifs that corresponding to the reference m^6^A sites among influenza A viruses. A total of 70,030 complete HA sequences that comprise all known HA subtypes (H1–18) collected over several years, countries, and affected host species were analysed on both mRNA and vRNA strands. The bioinformatic analysis revealed the highest degree of DRACHs conservation among all H1 sequences that clustered largely in the middle and in the vicinity to 3′ end with at least four DRACH motifs were conserved in all mRNA sequences. The major HA-containing subtypes displayed a modest DRACH motif conservation located either in the middle region of HA transcript (H3) or at the 3′ end (H5) or were distributed across the length of HA sequence (H9). The lowest conservation was demonstrated in HA subtypes that infect mostly the wild type avian species and bats. Interestingly, the total number and the conserved DRACH motifs in the vRNA were found to be much lower than those observed in the mRNA. Collectively, the identification of putative m^6^A topology provides a foundation for the future intervention of influenza infection, replication, and pathobiology in susceptible hosts.

## Introduction

Viral epitranscriptome is an emerging paradigm in the virus-host battle, which collectively describes all co- and post-transcriptional chemical covalent bonds installed on viral transcripts^1,2^. Among a handful of the chemical modification added to viral mRNAs, the methylation at N6-position of adenosine (m^6^A) exhibits superiority to dictate viral behaviour inside the cell, and regulate the fate of virus-infected cells predominantly the oncogenic viruses^3–10^. Moreover, the m^6^A residues have been known to regulate essential metabolic aspects of cellular RNA including stability, translation, splicing, nuclear export, and secondary structure^11–13^. Furthermore, critical attributes of life including embryonic development, fertility, immune modulation, and homeostatic functions were reported to be controlled by m^6^A^14–16^.

Despite m^6^A marks were initially identified to be incorporated in viral RNAs several decades ago, due to technological limitations at this time, topological and functional characteristics of m^6^A were not clear in viral-cell lifecycles^17–19^. In recent years, the ever-rising progress in epitranscriptome-wide sequencing technologies has been exploited to identify and relatively quantify m^6^A marks^20–24^. Hence highlights the unexplored aspects of m^6^A in host–pathogen interactions. The addition of the m^6^A marks on both cellular and viral RNA is governed by a complex group of nuclear methyltransferases (writers); these marks can be removed by another group of demethylases (erasers). Ultimately, a third group able to bind and generate a recognizable effect on the methylated transcripts (readers), all groups are collectively known as m^6^A machinery^9,25–27^. Installation of m^6^A is predominantly restricted to consensus sequence motif, the putative m^6^A sites significantly reported to be enriched in some 5′-DRA*CH-3′ sequences (where A* denotes the methylatable adenosine, D denotes A, G or U, R denotes A and G, and H denotes A, C or U)^24,28,29^.

Notably, the addition of m^6^A on cellular transcripts has been known to be enriched mostly in the coding sequence, 3′ untranslated region, and in the vicinity to stop codons^21^. Moreover, the epitranscriptome-wide analysis confirmed that the majority of methylation sites were conserved among murine and Homo sapiens, which emphasize the m^6^A sites were maintained throughout the evolutionary process via selection pressure^21,30,31^. Albeit, DRACH motifs are widespread all over cellular and viral transcriptomes, a small fraction only was reported to be methylated in vivo and the underlying mechanisms for the selection of certain DRACHs to accept methylation than others remain elusive^16,24^.

From the scanty viral epitranscriptome data, it is early to infer the accurate topology of m^6^A sites, mostly the highly variable viruses having various genotypes. Recently, m^6^A sites have been mapped in a single-nucleotide-resolution level in HIV-1, the data revealed that consensus m^6^A sites were relatively conserved across the tested isolates. Nonetheless, the genome of HIV-1 is characterized by a high degree of plasticity, highlighting that conservation of m^6^A sites implies many aspects in virus replication and/or subversion of immune-mediated responses^2,32^. Likewise, the m^6^A landscape has also been reported to be likely conserved and not restricted to certain strains in the ZIKA virus (ZIKV) model^33^.

Vis-à-vis influenza, epitranscriptomics analysis has identified multiple m^6^A residues across all the segmented transcripts^17^. Unravelling the viral methylome of A/Puerto Rico/8/34/Mount Sinai isolate has been recently mapped^4^. The latter study confirmed the positive impact of m^6^A on influenza virus replication and protein expression. Therefore, the determination of m^6^A sites among IAVs is most likely due in near future to intervene in influenza replication in a wide range of the affected host species. Although the m^6^A prediction software is available for cellular transcripts^34,35^, the m^6^A viral prediction software is still lacking and epitranscriptome-wide mapping to thousands of IAVs isolates limits its practicality. Herein, we aim to utilize publicly available meta-data on influenza virus in mapping m^6^A sites through the DRACH signatures and to predict the evolutionary conservation patterns among influenza A viruses.

## Results

### Identification of DRACH motifs in the reference HA for comparative analysis

**Figure 1:**
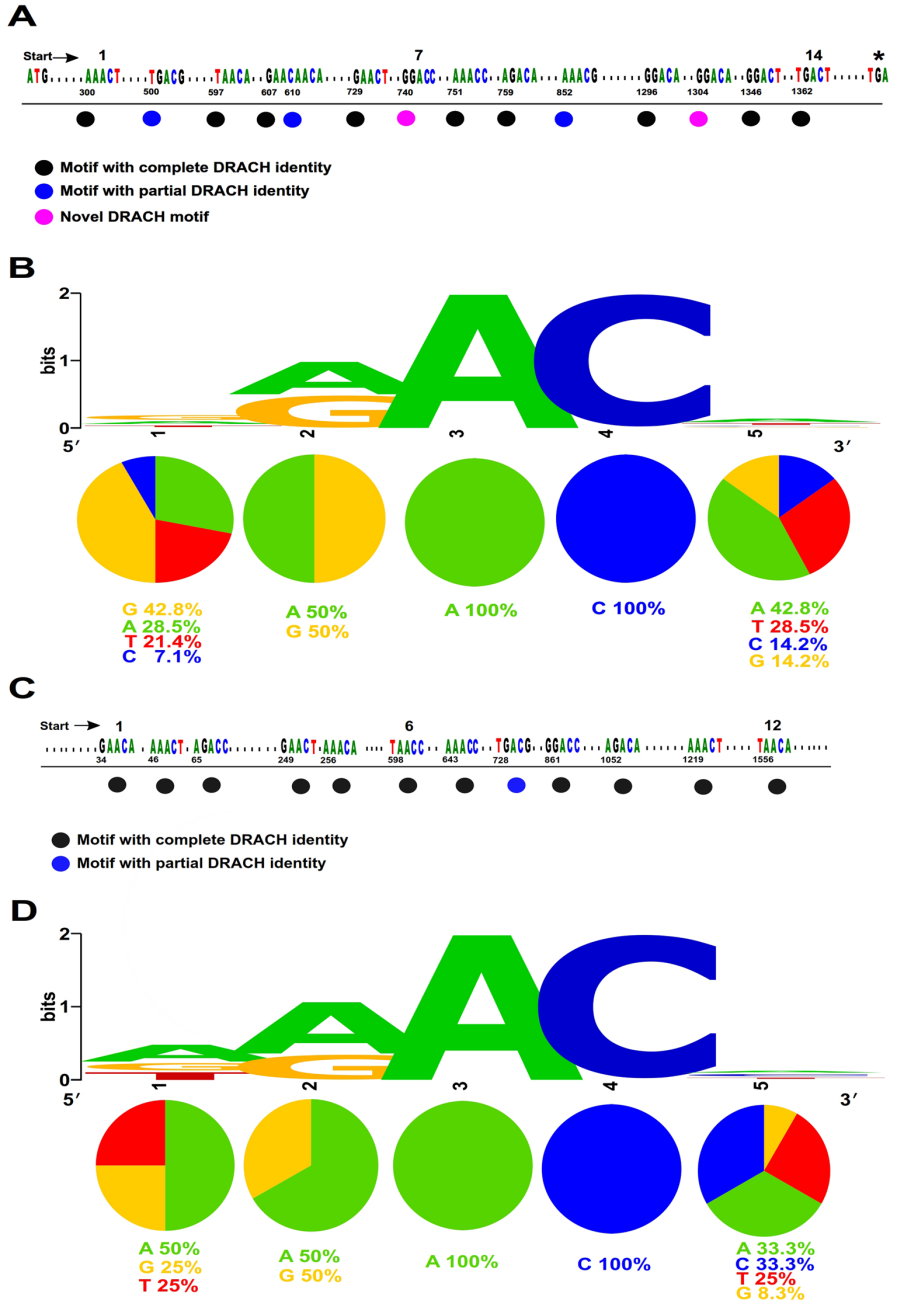
Locations and conserveness of the DRACH motifs utilized in this study. Schematic representation of locations of the putative motifs on the IAV-PR8 HA mRNA (**A**,**B**) and vRNA (**C**,**D**), accession no. AF389118, which coincident with the 8/9 m^6^A peaks identified in^4^ that are indicated by numbers. Motifs contain the complete, partial, and novel DRACH are indicated by coloured circles. (**B**,**D**) WebLogo-based diversity and/or conserveness of nucleotides in the proposed DRACH motifs in PR8 HA mRNA (**B**) and vRNA (**D**), one stack for each position in the sequence, the height of the stack indicates the sequence conservation at that position, the height of symbols within the stack indicates the relative frequency of each nucleotide at that position. The percent of each nucleotide in each position are indicated by coloured pie charts.

A total of 718,168 sequences were listed as IAVs sequences in the Influenza Research Database (IRD; as of December 15th, 2020). A total of 96,472 sequences were designated as full length HA sequences. From the default settings implemented in the IRD, duplicate HA sequences were removed to list only 70,030 unique HA sequences that were utilized for computational comparative analysis. Based on the previous H1N1 epitranscriptome-seq data^4^, eight and nine m^6^A sites were identified across the entire HA mRNA and vRNA, respectively (Fig. 1). However, these sites were not precisely identified due to their undescriptive and speculative characterization. Nonetheless, identified and functionally validated 12 out of 14 5′-RAC-3′ motifs corresponding to the 8 m^6^A sites have been mapped on mRNA^4^ (Fig. 1A,B). Similarly, twelve 5′-RAC-3′ motifs corresponding to the 9 m^6^A sites have been mapped on vRNA (Fig. 1C,D).

From the above-mentioned data, we adopted 14 5′-DRACH-3′ motifs for further analysis including the previously identified 12 5′-RAC-3′ motifs along with two additional 5′-DRACH-3′ motifs corresponding to the 8 previously mapped m^6^A sites on HA mRNA (motifs 7 and 12) (Fig. 1A,B). A total of 14 DRACH motifs were utilized as a base for subsequent conservation analysis across IAV HA mRNAs when compared with the reference A/Puerto Rico/8/34/Mount Sinai strain (accession number: AF389118) (Fig. 1A,B). The identified motifs were fairly distributed across the entire HA. However, clusters of motifs were in the middle of the gene and the vicinity to the 3′ end of HA mRNA (Fig. 1A). Although the reference HA sequence contains 14 5′-RAC-3′ identified motifs of interest, three did not match the wider 5′-DRACH-3′ sequences; motif 2 and 10 have incompatible H and motif 5 has incompatible D (Fig. 1A). The 14 identified wider motifs exhibited different conserveness and diversity according to the location of the nucleotide on the DRACH motif using WebLogo (Fig. 1B). Together, considering the 14 wider DRACH motifs might be more representative than the short RAC motifs for the computational conservation analysis of the putative m^6^A sites spanning HA mRNA of IAVs.

### Conservation pattern of the identified DRACH motifs among H1 subtype

To investigate the pattern of conservation of the identified DRACH motifs corresponding to the m^6^A sites among all IAVs HA mRNAs, a computational comparative analysis was generated to initially exploring the conservation of the identified 14 DRACH motifs among the known HA subtypes (i.e. H1, H2, … etc.), then explore the conservation of these motifs within the H1 subtype (i.e., H1N1, H1N2, … etc.) along with the affected host species as stated later. All analysis was compared with the reference A/Puerto Rico/8/34/Mount Sinai strain (accession number AF389118).

Concerning H1, the major subtype of IAVs that affects predominantly human and swine and harbouring the highest number of HA sequences listed on IRD (36%). A total of 25,576 entire unique HA sequences were analyzed. We identified 40 typical DRACH motifs across the complete consensus H1 sequence (Table 1). The H1 consensus sequence was aligned with the reference HA mRNA. After a comparison of the identified 14 DRACH motifs and counting the number of matches of each motif of interest with the H1 subtype, sequences revealed that 6 out of the 14 DRACH motifs were found to be highly conserved. The conservation percentage was 99% in 7th, 12th, and 13th motifs, and 95%, 90%, and 85% in 6th, 9th, and 11th motif, respectively (Table 1, Supplementary Fig. 1).

**Table 1:** Summary of conserved DRACHs distributions among all IAVs HA mRNAs.

mRNA	Seq no.^a^	DRACH^c^	Motif1	Motif2	Motif3	Motif4	Motif5	Motif6	Motif7	Motif8	Motif9	Motif10	Motif11	Motif12	Motif13	Motif14
H1N1 PR8^b^	1	40	✓	✓	✓	✓	✓	✓	✓	✓	✓	✓	✓	✓	✓	✓
H1	25,576	40						✓ 95%	✓ 99%		✓ 90%		✓ 85%	✓ 99%	✓ 99%	
H2	618	36	✓ 65%			✓ 95%			✓ 99%		✓ 50%			✓ 99%	✓ 99%	✓ 50%
H3	23,286	42		✓ 90%					✓ 99%	✓ 90%	✓ 90%					✓ 85%
H4	1646	51		✓ 65%		✓ 65%			✓ 99%	✓ 50%			✓ 65%			✓ 85%
H5	5472	34							✓ 99%	✓ 75%				✓ 99%	✓ 99%	✓ 75%
H6	1836	40			✓ 85%					✓ 75%				✓ 99%	✓ 99%	
H7	2237	45						✓50%			✓50%		✓ 95%			✓50%
H8	168	48		✓ 95%								✓ 85%	✓ 50%		✓ 95%	✓65%
H9	6408	43		✓ 99%						✓90%					✓ 99%	
H10	983	45							✓95%	✓50%			✓ 95%			
H11	781	38	✓ 50%			✓ 75%			✓95%	✓75%			✓ 90%		✓ 90%	
H12	337	46		✓ 90%		✓ 75%				✓85%			✓ 85%		✓ 95%	
H13	409	43													✓ 95%	
H14	35	44							✓ 100%							✓ 100%
H15	16	44											✓ 50%			
H16	217	41													✓ 99%	
H17	2	41											✓ 100%		✓ 100%	
H18	2	47				✓ 100%									✓ 100%	

^a^The number of HA sequences listed on IRD and analyzed here in this study.

^b^The reference influenza A virus strain utilized in this study, A/Puerto Rico/8/34/Mount Sinai, HA accession no. AF389118.

^c^The total number of the possible DRACH motifs located in the consensus sequence in each subtype.

Of particular note, the conservation percentage of the 5th motif was recorded in 85% of the H1 sequences, however, did not consider a conserved motif (putative m^6^A site) due to lack of the complete DRACH sequence identity. Additionally, remaining motifs exhibited loss to any/all nucleotide(s) when compared with the reference sequence (Supplementary Fig. 1). Furthermore, the conserved motifs are clustered in two regions as that identified in the HA reference transcript. Collectively, a high degree of DRACH motifs showed conservation among all H1 sequences and clustered largely in the middle and in the vicinity to 3′ end of H1 mRNAs.

### Conservation pattern of the identified DRACH motifs among H3, H5, and H9 subtypes

Likewise, we investigated the pattern of conservation of identified the DRACH motifs in H3, H5, and H9 subtypes, which enclose 23,286, 5472, 6408 unique HA mRNA sequences, respectively (collectively represent about 50% of HA sequences listed on IRD). These subtypes infect mainly humans, swine, equine, and avian species in various geographic distributions all over the world. The conservation pattern of DRACH motifs of interest of H3 subtype revealed partial or complete loss of 9 DRACH motifs compared with the H1N1 PR8 HA reference, however, a higher degree of conservation identified in 5 motifs; 2, 7–9, and 14 with a conservation percentage 85–99% as indicated in (Table 1, Supplementary Fig. 2A). Regarding the H5 consensus HA sequence, A total of 5 DRACH motifs were also found to share conservation with the reference HA; two motifs were conserved in the middle cluster of H5 transcripts; motif-7 and -8 in 99 and 75% of all H5 transcripts, respectively. Furthermore, the last three DRACH motifs were identified to be conserved as well (conservation percent 75–99) (Table 1, Supplementary Fig. 2B).

The H9 subtype displayed only 3 widely separated conserved motifs; near 5′ end, in the middle region, and near 3′ end with the highest degree of conservation (90–99%) among all major HA subtypes. Notably, 50% of H9 sequences at motif 14 contain RAC motif, however, lack of a complete DRACH sequence was noticed to exclude this motif from the conservation pattern (Supplementary Fig. 2C). Together, all major HA subtypes were identified to have partial DRACH sequence conservation compared with the reference sequence that clustered either in the middle region of HA transcript (H3), 3′ end of HA mRNA (H5), and distributed all over the HA sequence (H9).

### Conservation pattern of the identified DRACH motifs among other subtypes

**Figure 2:**
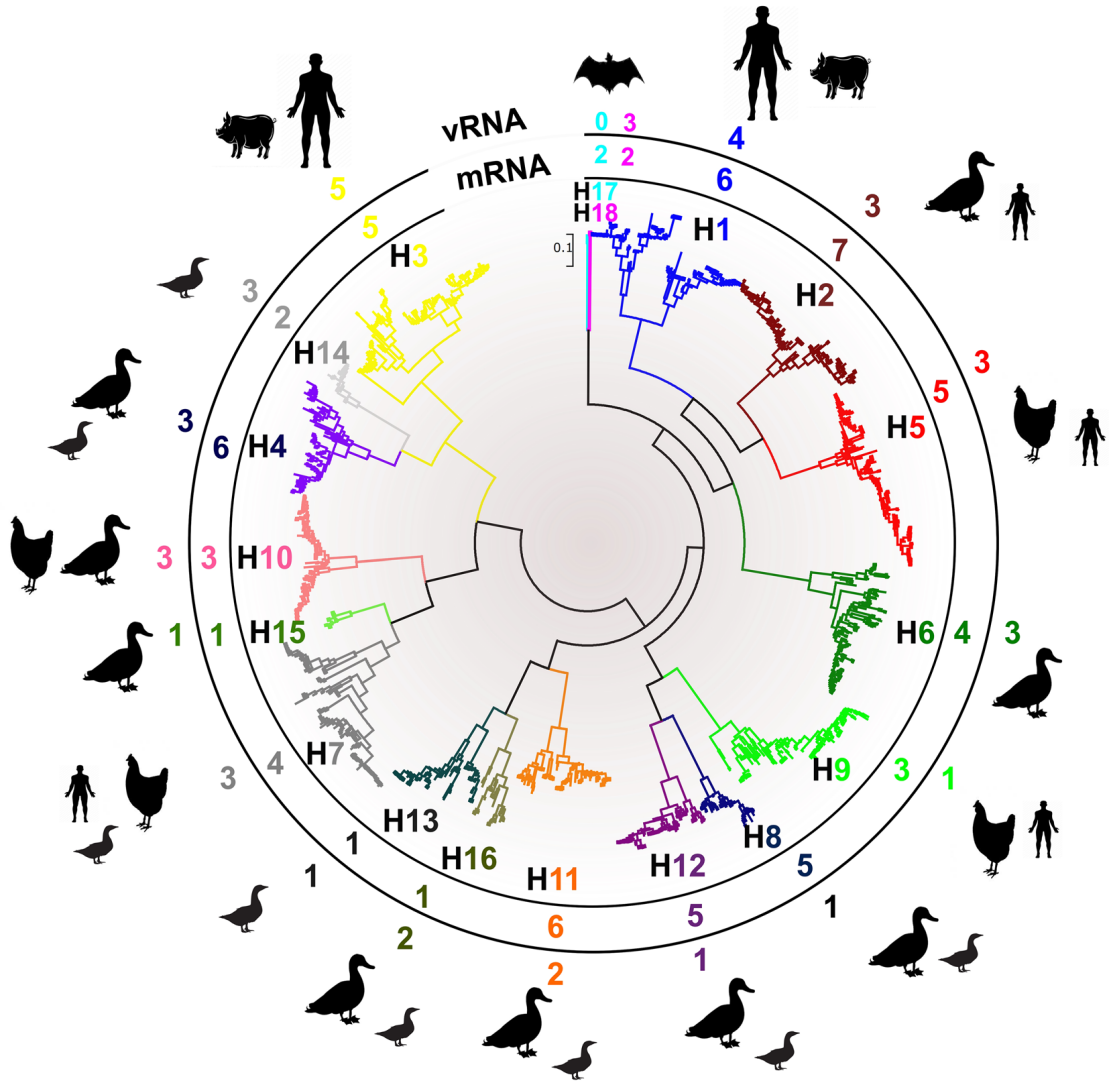
Summary of conserved DRACHs among all HA subtypes of IAVs. From the centre, the HA cladding system is represented by a maximum-likelihood tree contain representing sequences of each subtype. The numbers of conserved DRACH motifs to each HA subtype that located on either vRNA or mRNA are indicated. The highly susceptible species affecting each subtype are indicated.

Similarly, we tested the conservation pattern of the identified DRACH motifs between remaining HA sequences (i.e. the lower HA number containing subtypes) that collectively enclose 13% of all HA sequences recorded in IRD. These subtypes infect mainly the wild avian species (H10–16), human (H2 and H7), and bats (H17 and H18). The unique HA sequence numbers and the number of conserved DRACH motifs in each subtype were shown in (Fig. 2). Each HA subtype consensus sequence was aligned and compared with reference HA of interest.

Regarding the H7 subtype, the highest conserved DRACH was detected in the 11^th^ motif (95% among 2237 sequences), whilst, H4 (1646 sequences) witnessed the highest conservation in 7th and 14th DRACH. Subtypes affecting mostly avian species (H10-16) revealed DRACH motif conservation near 3′ end mostly in 11th and 13th. Markedly, bat IAV HA sequences (i.e. H17 and H18) revealed conservation at motif 13 (Table 1). It is important to note that infrequent conserved motifs were presented in this group of HA sequences; motif-1 in H11 and H2, motif-3 in H6, and motif-10 in H8 with varying conservation percentage as indicated in (Table 1). This group of HA subtypes demonstrates the lowest number of DRACH motif conservation. Collectively, the 3′ end DRACH motifs were found to be conserved in wild avian and bats sequences. Motifs 13, 11, and 7 were identified to be the highest conserved among all analyzed HA sequences (Table 1).

### Conservation pattern of the identified DRACH motifs among various host species and viruses within the subtypes

To deeply investigate the conservation pattern of DRACH motifs among various host species, pathogenicity, geographic locations, and further subtyping. A detailed DRACH conservation analysis was generated within subtypes implicated with major human and avian infections. H1N1 sequences comprise more than 80% of all H1 sequences recorded in IRD. H1N1 maintained the 6 previously identified conserved DRACH motifs of all H1 sequences (Table 2). To explore the effect of DRACH sequences conservation pattern on pathogenicity, the pandemic H1N1 (pH1N1) sequences were compared with non-pandemic sequences. Human H1N1 sequences revealed that the pH1N1 sequences have no clear differences from identified the 6 conserved DRACHs of all H1 sequences in all affected countries. In contrast, 50% of the non-pH1N1 sequences gain an additional motif (i.e. motif 8). Moreover, compared to humans, swine H1N1 sequences exhibited loss of motif-11 and gain of motif-14. Furthermore, H1N2 sequences lost motif-9 in both human and swine sequences. Markedly, motifs 6, 7, 12, and 13 were conserved regardless of viruses, species, pathogenicity, and geographic locations (Table 2).

**Table 2:** Summary of conserved DRACHs among various species and viruses of H1 mRNAs.

mRNA	Seq no	Motif2	Motif6	Motif7	Motif8	Motif9	Motif11	Motif12	Motif13	Motif14
H1	25,576		✓ 95%	✓ 99%		✓ 90%	✓ 85%	✓ 99%	✓ 99%	
Human H1N1	16,471		✓ 95%	✓ 99%		✓ 90%	✓ 90%	✓ 99%	✓ 99%	
Pandemic H1N1	10,993		✓ 99%	✓ 99%		✓ 99%	✓ 99%	✓ 99%	✓ 99%	
Non-pandemic H1N1	5490		✓ 95%	✓ 99%	✓ 50%	✓ 65%	✓ 99%	✓ 99%	✓ 99%	
Swine H1N1	4366		✓ 50%	✓ 95%		✓ 95%		✓ 99%	✓ 99%	✓ 65%
Human H1N2	43		✓ 95%	✓ 100%	✓ 90%		✓ 99%	✓ 99%	✓ 99%	
Swine H1N2	3614	✓ 50%	✓ 90%	✓ 95%	✓ 65%		✓ 75%	✓ 99%	✓ 99%	✓ 65%

**Figure 3:**
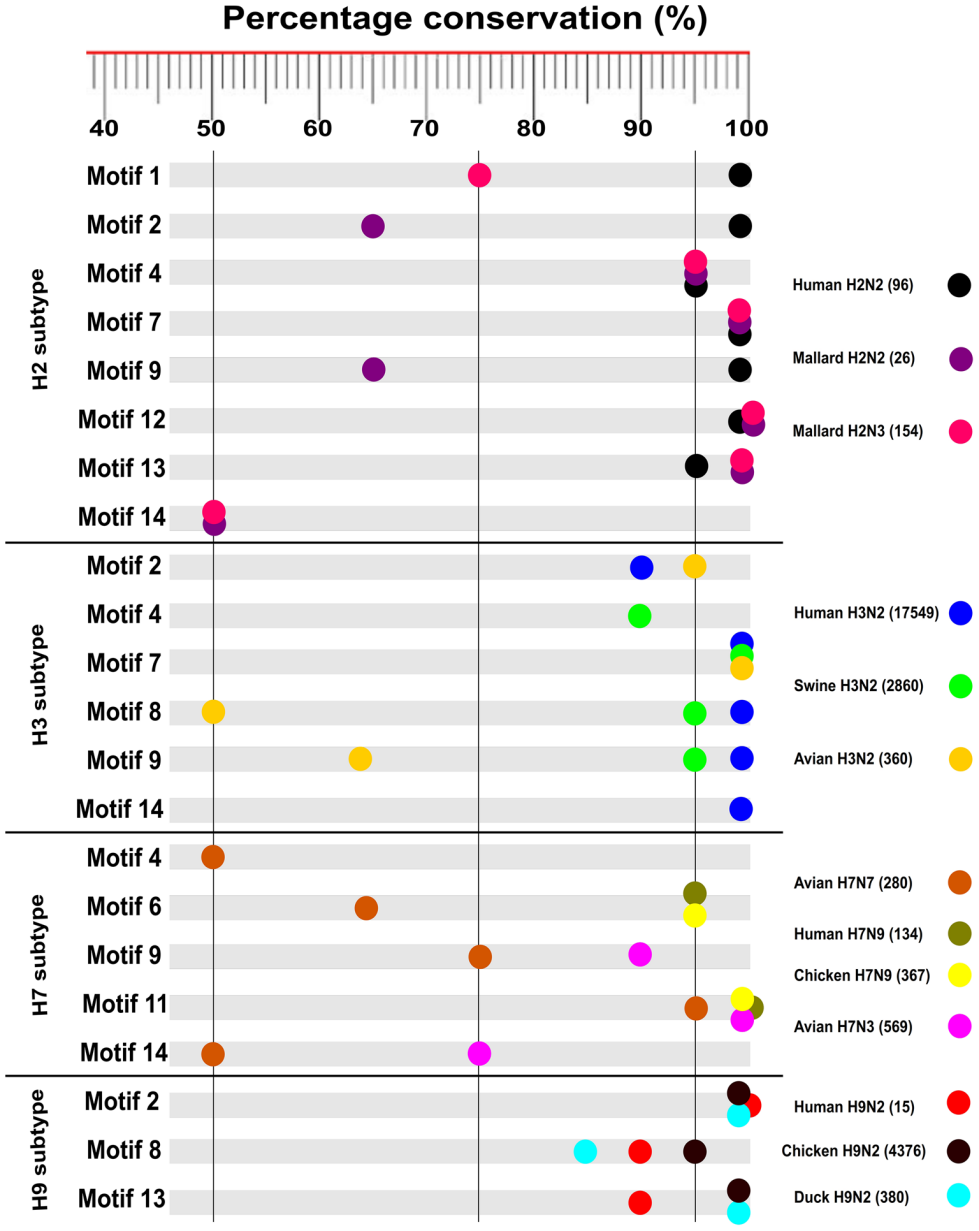
Summary of conserved DRACH motifs among some IAVs HA mRNAs. Conserved motifs, virus, host species, and the number of sequences listed on IRD are indicated. Conservation percent is indicated on the upper side.

**Figure 4:**
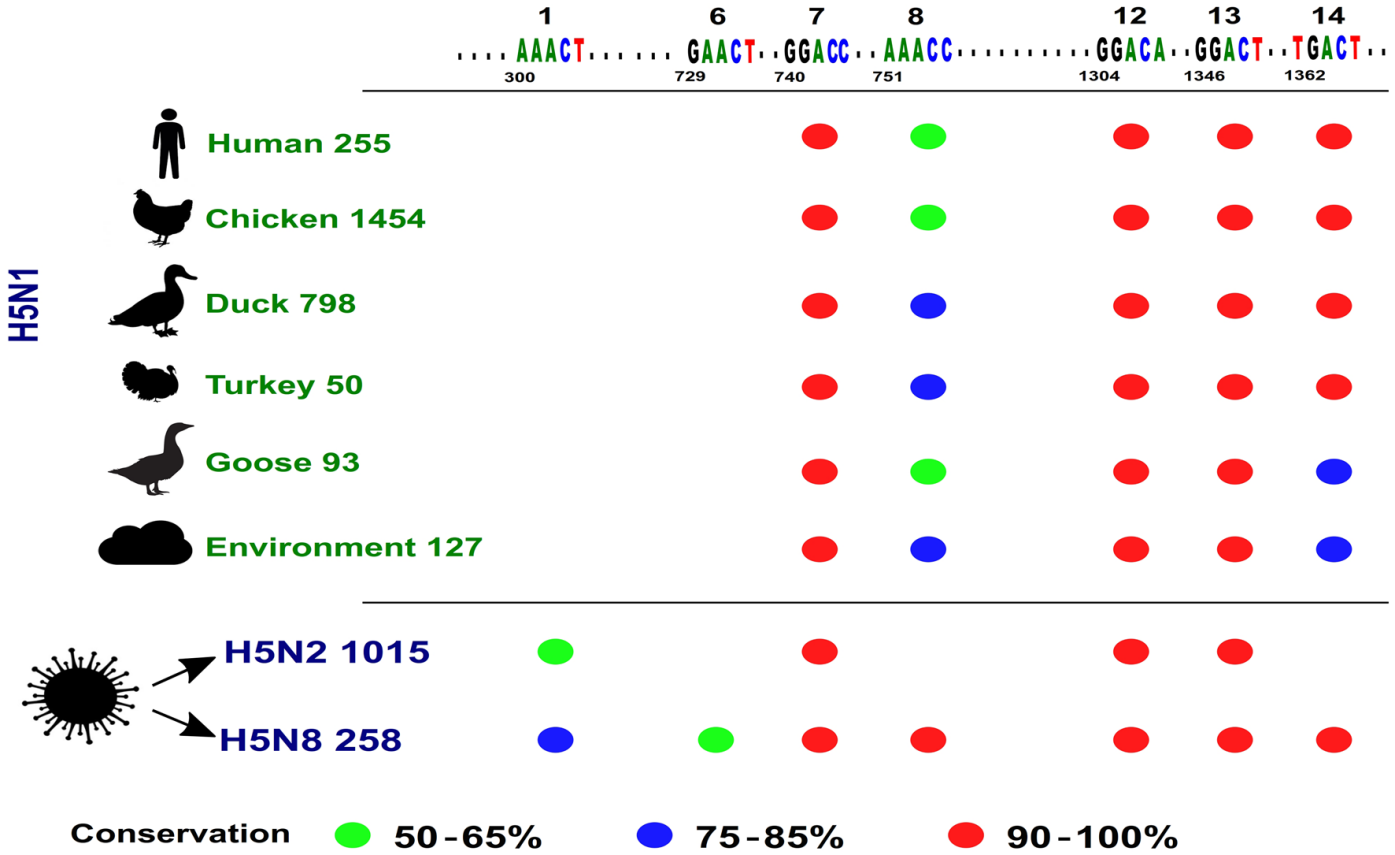
Summary of conserved DRACH motifs among the H5 mRNA. Conserved motifs, virus, host species, and the number of sequences listed on IRD are indicated. The conservation percent is indicated by coloured circles.

Similarly, compared to the H2 consensus sequence, human H2N2 sequences lost motif-14, while, mallard, the most common species infected by H2 sequences, lost motif-1 in H2N2 sequences (Fig. 3). Furthermore, human H3N2 sequences the sole to contain motif-14, compared to swine and avian sequences (Fig. 3). Markedly, human influenza sequences descended from zoonotic (chicken) origin; H5N1, H7N9, and H9N2 maintained the same conserved motifs of the original chicken viruses (Figs. 3, 4). Collectively, DRACH motifs seem likely to be virus-specific rather than host species-, pathogenicity-, and geographic distribution-specific.

### Conservation patterns of identified DRACH motifs among IAVs HA vRNA

Considering IAVs are negative-sense single-stranded RNA viruses^36^, we sought to investigate the level of conservation of DRACH motifs in the genomic sequence as well. Based on the previous H1N1 meta-data^4^, a total of nine putative m^6^A sites were identified across the entire HA vRNA which were not described before. These novel 9 m^6^A sites corresponding to 12 RACs which have been identified and functionally validated in the vRNA of the reference A/Puerto Rico/8/34 strain (Accession Number: AF389118)^4^. These 12 motifs were adopted for further analysis on HA vRNA among IAVs in this study. Likewise the m^6^A motifs were fairly distributed across the entire length of the mRNA of IAVs (Fig. 1C). We compared these 12 motifs among all HA subtypes. We noticed that one out of the 12 motifs did not match with the wider DRACH (i.e. motif 8) sequence. Intriguingly, this motif was found to be non-conserved in the H1 subtype (Table 3).

**Table 3:** Summary of conserved DRACHs distributions among all IAVs HA vRNAs.

vRNA	Seq no.^a^	DRACH^c^	Motif1	Motif2	Motif3	Motif4	Motif5	Motif6	Motif7	Motif8	Motif9	Motif10	Motif11	Motif12
H1N1 PR8^b^	1	27	✓	✓	✓	✓	✓	✓	✓	✓	✓	✓	✓	✓
H1	25,576	29			✓ 95%			✓ 90%				✓ 95%		✓ 90%
H2	618	26	✓ 50%						✓ 50%			✓ 65%		
H3	23,286	25					✓ 85%	✓ 75%	✓ 85%	✓ 95%			✓ 95%	
H4	1646	26	✓ 95%				✓ 75%			✓ 99%				
H5	5472	27				✓ 75%	✓ 75%		✓ 50%					
H6	1836	23	✓ 85%	✓ 95%								✓ 50%		
H7	2237	26						✓ 75%	✓ 50%	✓ 95%				
H8	168	26							✓ 95%					
H9	6408	26					✓ 85%							
H10	983	19			✓ 95%			✓ 85%					✓ 50%	
H11	781	42					✓ 90%		✓ 75%					
H12	337	25	✓ 99%											
H13	409	24					✓ 65%							
H14	35	19	✓ 100%		✓ 100%		✓ 100%							
H15	16	21					✓ 100%							
H16	217	26						✓ 75%					✓ 75%	
H17	2	17												
H18	2	17	✓ 100%				✓ 50%		✓ 100%					

^a^The number of HA sequences listed on IRD and analyzed here in this study.

^b^The reference influenza A virus strain utilized in this study, A/Puerto Rico/8/34/Mount Sinai, HA accession no. AF389118.

^c^The total number of the possible DRACH motifs located in the consensus sequence in each subtype.

Albeit total DRACH motifs in the vRNA in each subtype were considerably much lower compared to the mRNA of the same subtype (about 50%). A considerable level of conservation was noticed in the 12 DRACH motifs of interest in each subtype (Table 3). The highest conservation was noticed in H1 and H3 subtypes. Unlike the mRNA, H9 was reported to contain only one conserved motif (i.e. motif 5) in the HA vRNA. Additionally, the HA subtypes that infect mainly wild bird species and bats were noticed to have only a few DRACH motif conservation similar to that of the mRNA. Moreover, motifs 5, 7, and 11 recorded the highest motifs that witnessed conservation among the vRNAs of HA subtypes. Whereas motifs 2, 4 and 12 shown to have lowest conservation among IAVs.

To deeply investigate the conservation pattern of DRACH motifs among various host species, pathogenicity, geographic locations, and for further subtyping in vRNA, a detailed DRACH conservation analysis was generated within the H1 subtype. We noticed that the H1N1 maintained four previously identified conserved DRACH motifs in all H1 sequences (Table 3; Supplementary Table 1). Additionally, to explore DRACH sequences conservation pattern on pathogenicity, the pandemic H1N1 (pH1N1) sequences compared with non-pandemic sequences. Human H1N1 sequences revealed that the pH1N1 sequences have no clear differences from the identified 4 conserved DRACHs of all H1 sequences in all affected countries. In contrast, 50% of the pH1N1 sequences gain an additional motif (i.e. motif 2) on vRNA. Additionally, the swine H1N1 gained additional motif compared to humans. Interestingly, H1N2 sequences witnessed much higher conserved DRACH than that of H1N1 sequences including 9 motifs in human H1N2 and 8 in swine. Markedly, 4 motifs were conserved regardless of viruses, species, pathogenicity, and geographic locations (Supplementary Table 1). Collectively, compared to the mRNA, the vRNA witnessed a lower conservation level in both the total and the DRACH Motifs of interest, the highest conservation was detected in the major HA− containing subtypes and much lower in HA− subtypes affecting wild avian species and bats, and H1 contain 4 conserved motifs in almost all sequences.

## Discussion

Influenza A viruses are single-stranded octameric segmented RNA belonging to the *Orthomyxoviridae* family. IAVs are characterized by high mutation rates with the hemagglutinin gene (HA) confirmed to have the record highest mutation rate. HA gene is responsible for the remarkable genetic plasticity among all remaining IAVs genes. The high mutation rate along with the reassortment of IAVs segmented nature can lead to the emergence of pandemics. Additionally, IAVs can infect a wide range of host species including humans, animal, and avian species suggesting the likely roles to cross species barriers and causing zoonotic fatal infections^36–38^.

The epigenetic m^6^A mark is the most abundant internal chemical modification installed onto both cellular and viral mRNA that dictate various cell behaviours and the fate of the virus-host interaction^2,39^. The significance of m^6^A has been highlighted by the fact that this modification is evolutionarily conserved among vertebrates^31^, besides, a high degree of conservation in the majority of m6A machinery has recently been confirmed among vertebrates^9^. Furthermore, the overall inhibition of m^6^A has various detrimental effects on animals' life^13^. Therefore, the determination of the characteristic features and topology of viral m^6^A is of paramount importance to modulate the outcome of the virus-host battle. However, the utilized methods to precisely determine the locations of m^6^A marks remain limited in viral epitranscriptomics, laborious, and non-cost-effective to investigate thousands of virus strains. Moreover, the highly evolving nature of viruses could complicate this notion.

Among viruses, influenza was the first model identified to express mRNAs harbour multiple m^6^A marks in the 1970s^17,19^. These earlier studies identified 24 m^6^A sites across the entire segmented genome with eight of which detected in the HA mRNA via biochemical analysis. In agreement with recent high-throughput sequencing data using PAR-CLIP and PA-m^6^A-seq, eight/nine prominent m^6^A sites were identified across the HA mRNA/vRNA of H1N1^4^, respectively. However, the adopted approaches of sequencing did not provide a precise determination of the methylated adenosines at single-nucleotide resolution. In this milieu, we utilized the commonly used consensus sequence where m^6^A marks are installed; the DRACH motif that was described in cellular transcriptome-wide maps^21,30^. Moreover, the m^6^A individual-nucleotide-resolution cross-linking and immunoprecipitation (miCLIP) sequence data revealed that m^6^A were markedly more prevalent in the DRACH sequence context^24^. Furthermore, several loss-of-function experiments have tested the functionality of m^6^A marks through mapping along with synonymous ablation of the DRACH motifs from both cellular^40^ and viral transcripts^4,5,41^. Therefore, we exploited the DRACH motif to figure the pattern of conservation among IAVs through comparative analysis. This wider motif could narrow the window against the evolving nature of the IAVs than the shorter RAC motif.

It is plausible that the number of RAC/DRACH motifs could be higher than the number of the actual m^6^A sites, as multiple close motifs might be present underneath the m^6^A detection peak mostly in the non-single-nucleotide-resolution approaches, which could be a challenge to confirm the methylated adenosines. This is a characteristic feature for m^6^A additions to cellular transcripts^24^. Regarding influenza, the introduction of 12 out of 14 synonymous mutations to the short RAC motifs corresponding to the eight m^6^A sites on mRNA has been performed^4^. The two other RAC motifs have not been mutated without alteration of the amino acid code (identified here as motif-7 and 12, Fig. 1A). Herein, these 14 motifs were used as a base for conservation analysis. Three RAC motifs did not match with the wider DRACH identity, thus could be highly unlikely real m^6^A sites. Surprisingly, our comparative analysis on mRNA revealed that these 3 motifs had the lowest conservation among IAVs (Table 1). Notably, m^6^A methyltransferases are strictly favouring installing m^6^A on highly conserved consensus sequence^42^.

A bioinformatic approach is adopted here to predict the highly conserved DRACH motifs that corresponding to m^6^A sites across HA mRNA/vRNA. We focused only on the HA genes as it encodes the highly variable major viral structural glycoprotein^43^ and the HA gene only was fully mapped in previous literature^4^. The IRD was adopted to decipher the conservation patterns of DRACH motifs of interest utilizing its implemented bioinformatic algorithms between approximately 70,000 complete HA sequences^44^. Six out of the 14 full DRACH motifs were identified to share conservation of 85–99% of the H1 subtype sequences (more than 25,000 HA sequences) compared with the reference H1N1 A/Puerto Rico/8/34/Mount Sinai. This high level of conservation highlights that these putative m^6^A sites are not PR8 specific among a highly variable virus generally and HA specifically^38^. Furthermore, clustering of the conserved motifs in the middle and vicinity to 3′ end suggests an underlying 3D RNA structure, mRNA folding, and stability regulatory function as previously described in the HIV-1 model^2,32^. Notably, the middle cluster DRACHs are located near the junction between HA1 and HA2 fragments (Supplementary Fig. 2). Albeit, some variations were observed through in-depth comparative analysis demonstrated by DRACH gain or loss among various host species affected by viruses of H1 subtype, four motifs were conserved whatever the affected host species, virus collection date, and geographic location (Table 2), similarly, the same conservation pattern was found in the HA vRNA among IAVs (Table 3, Supplementary Table 1).

Likewise, the conservation of the DRACH motifs of interest in HA mRNAs of H3, H5, and H9 subtypes (collectively comprising more than 50% HA sequences) were confirmed. Of note, the HA cladding system categorizes H1, H5, and H9 sequences as clade I, whilst H3 sequences belonging to clade II based on the entire HA sequence phylogeny^45^ (Fig. 2). Nonetheless, H3 HA sequences are still harboring at least 85% conservation of not only the middle cluster DRACH motifs namely 7–9 that constitute a part of the HA1 fragment, but conservation of motif-14 at the HA2 fragment was also noticed. Moreover, the available H5 and H9 HA sequences maintain a pattern of conservation to those DRACH motifs of both the middle region and near the 3′ end of HA.

In a trial to address influenza pathogenicity and m^6^A sites correlations, the in-depth comparative analysis showed that the low pathogenic avian influenza virus of chicken-H5N2 harbours lower conserved DRACH motifs compared with highly pathogenic avian influenza viruses of chicken-H5N1 and -H5N8^46–49^. In contrast to this finding, the pandemic H1N1 sequences show lower conserved DRACH motifs compared with non-pandemic sequences^50^ (Table 2). Interestingly, on mRNA human influenza HA sequences that originated from avian species H5N1, H9N2, and H7N9 share the same DRACH motifs of the original chicken viruses (Fig. 3). Same finding was confirmed in vRNA as well between human influenza HA sequences that originated from avian species H5N1, H9N2, and H7N9 (Data not shown). Considering the enhanced pathogenicity of zoonotic viruses as confirmed by mortalities after transmission to humans^38^ compared with low pathogenic nature in chicken (i.e. H9N2, H7N9) seems peculiar to connect to pathogenicity (Fig. 3). Furthermore, conservation of the same DRACH motifs from various host species (i.e. human, chicken, turkey, duck, goose, and environmental isolates in the H5N1 model) was observed (Fig. 4). Collectively, it seems that the conserved DRACH motifs (the putative m^6^A sites) pointing to be virus-specific predominantly rather than to be host species-, pathogenicity-, clade-, and geographic-specific even in various forms of RNA to the same virus model. The same findings were noticed in HIV-1 and Zika virus models^2,32,33^. However, large-scale functional studies warrant further investigation to support this notion.

The remaining IAVs subtypes that infect mostly the wild avian species, humans, and bats (collectively comprise 13% of the HA sequences listed on IRD) harbour the infrequent conserved DRACH motifs; motif-1 in H2 and H11, motif-3 in H6, and motif 10 in H8. The scanty number of HA sequences identified so far per each subtype could be the cause of this finding, and higher numbers are required to give an appropriate interpretation. Nonetheless, the highest conserved DRACH motifs among the remaining subtype HA sequences; motifs-13, -11, and 7 are still observed (Table 1). These observations highlight that the putative m^6^A sites of this group of IAVs subtypes are conserved regardless of the low pathogenicity in the affected host species^51^ and might be connected to the influenza virus itself. However, functional testing is still required. The massive numbers of HA sequences analysed here revealed that the lowest DRACH motifs to encounter conservation are motifs 5 and 10. It can be seen that these motifs in the PR8 reference sequence lack the full DRACH sequence identity on mRNA. Likewise the case in mRNA, motif 8 on HA vRNA that lacks the full DRACH sequence identity of PR8 were found be less conserved among H1 subtype (about 25,000 sequences). Notably, unlike PR8, motif 2 was found to encounter conservation in 5 HA subtypes which harbour the correct DRACH sequences identity (Table 1). Intriguingly, cellular transcripts prediction software SRAMP^34^ also confirmed that 5 DRACHs are shared in PR8 conserved motifs described here; 6, 7, 11, 12, and 13 (data not shown). The 13th DARCH motif recorded the highest confidence site. However, other sites predicted in SRAMP did not match mostly with the publicly available PR8 mapped sites.

Reduction of the conserved DRACH motifs of interest among the newly emerged influenza viruses in bats the H17 and H18 and most influenza viruses characterized in wild birds (H13–16) remains in question. Notably, all HA subtypes share comparable numbers of total DRACH sites on the entire HA consensus sequences (36–47 DRACH sites) (Table 1). Additionally, the lower number of the total and DRACH sequences of interest in vRNAs compared with mRNAs might emphasize the importance of this signature on the translation and stability to mRNA, however, further functional validations are in need in near future. Further analysis to decipher the association with the level of m^6^A sites and the replication efficiency and pathogenicity in these species is required. Ultimately, future efforts to precisely map m^6^A sites on various viral transcripts coupled with the targeted mutagenesis of m^6^A sites will be a valuable tool to modulate the fate of the virus-host battle, a step toward the intervention of critical pathogens. Additionally, will assist in the generation of viral m^6^A prediction software to unveil critical aspects in infection cell biology.

## Materials and methods

### Determination of the reference viral transcript for comparative analysis

Based on the public IAV epitranscriptome-wide profiling data^4^, a comparative assessment was performed. Briefly, they utilized two different sequencing techniques to map various m^6^A sites across H1N1 A/Puerto Rico/8/34/Mount Sinai isolate^20,23^. Based on m^6^A-seq, a total of eight/nine m^6^A sites were identified across the entire hemagglutinin (HA) mRNA and vRNA, respectively (NCBI Accession # AF389118) and functionally confirmed their sequence analysis through synonymous mutation of 12 out of 14 short 5′-RAC-3′ motifs corresponding to the 8 identified m^6^A sites on the mRNA and the 9 identified m^6^A sites on the vRNA^4^ because the adopted sequencing approaches were not sensitive to identify the m^6^A sites in a single-nucleotide-resolution level. Herein, we utilized both the mRNA- and vRNA-HA from A/Puerto Rico/8/34/Mount Sinai as references for further computational comparative analysis to test the conservation patterns of putative m^6^A sites among all HA subtypes of IAVs.

### HA dataset collection

Nucleotide sequences of the entire HA gene of all IAVs were retrieved from the NIAID Influenza Research Database (IRD www.FluDB.org). We adopted in the settings that all HA subtypes from H1 through H18 were separately selected. In each HA subtype all possible neuraminidase (NA) subtyping, host species, date range, and geographic distribution were included. For further analysis, certain settings to include specific viruses within subtype(s) (i.e. H1N1, H1N2… etc.) or specific host species were adopted as stated later. Laboratory strains and duplicated sequences were excluded from the computational analysis. The influenza H1N1 A/Puerto Rico/8/34/Mount Sinai (AF389118) was added to each HA subtype to serve as a reference strain for subsequent comparison.

### Multiple sequence alignment, generation of consensus sequences, and identification of the conserved DRACH sites

Multiple sequence alignments (MSA) were generated to the long open reading frame of each tested HA subtype (i.e. H1-H18, separately) using the MUSCLE algorithm of the MSA tool implemented in IRD^44^. The computed and visualized MSA results together with the derived consensus sequences downloaded in FASTA format. Geneious software (v9.1.4) was used to further confirm the alignment and the generated consensus sequences utilizing the same MSA algorithm^52^. The latter software can provide consensus sequences based on the given threshold frequency (TF) percentage and display the non-consistent bases as IUPAC degenerate nucleotides. Each HA subtype consensus sequence was aligned with the reference H1N1 HA sequence using the Clustal W algorithm, Lasergene software package, version 3.18 (DNASTAR, Madison, WI) for identification of mutation hotspots, and prediction of nucleotide changes, then visualized using BioEdit program, v7.2.5 (Ibis Biosciences, Carlsbad, CA)^53^. A putative m^6^A site was considered specific and conserved if has the full 5′-DRACH-3′ motif sequence (D = A, G or T; R = A or G; H = A, C or T) when compared with the reference HA sequence and presented by the lowest conservation percentage of the total number sequences of the analysed subtype.

### Conserveness of DRACH motifs

Conserveness and diversity of all putative motifs were identified using WebLogo https://weblogo.berkeley.edu/logo.cgi^54^, in which DRACH motifs (5 nucleotides) presented as stacks, each stack for a position in the sequence, the height of the stack indicates the sequence conservation at that position, the height of symbols within the stack indicates the relative frequency of each nucleotide at this position.

## Supplementary Information

Supplementary Information.
