# Supplementary material for: Evolutionary conservation of the DRACH signatures of potential N6-methyladenosine (m6A) sites among influenza A viruses
Source: Sci Rep. 2021 Feb 25;11:4548. doi: 10.1038/s41598-021-84007-0 (PMC7907337; doi:10.1038/s41598-021-84007-0)
Supplement: Supplementary file 1 — Supplementary Information. [file 41598_2021_84007_MOESM1_ESM.pdf]

Evolutionary Conservation of the DRACH signatures of potential N6-methyladenosine (m^6A) sites Among Influenza A Viruses

Mahmoud Bayoumi and Muhammad Munir*

Division of Biomedical and Life Sciences, Lancaster University, Lancaster, LA1 4YG, United Kingdom

Correspondence:
Email: muhammad.munir@lancaster.ac.uk; Tel: +44 (0)1524 595083

Supplementary figures:

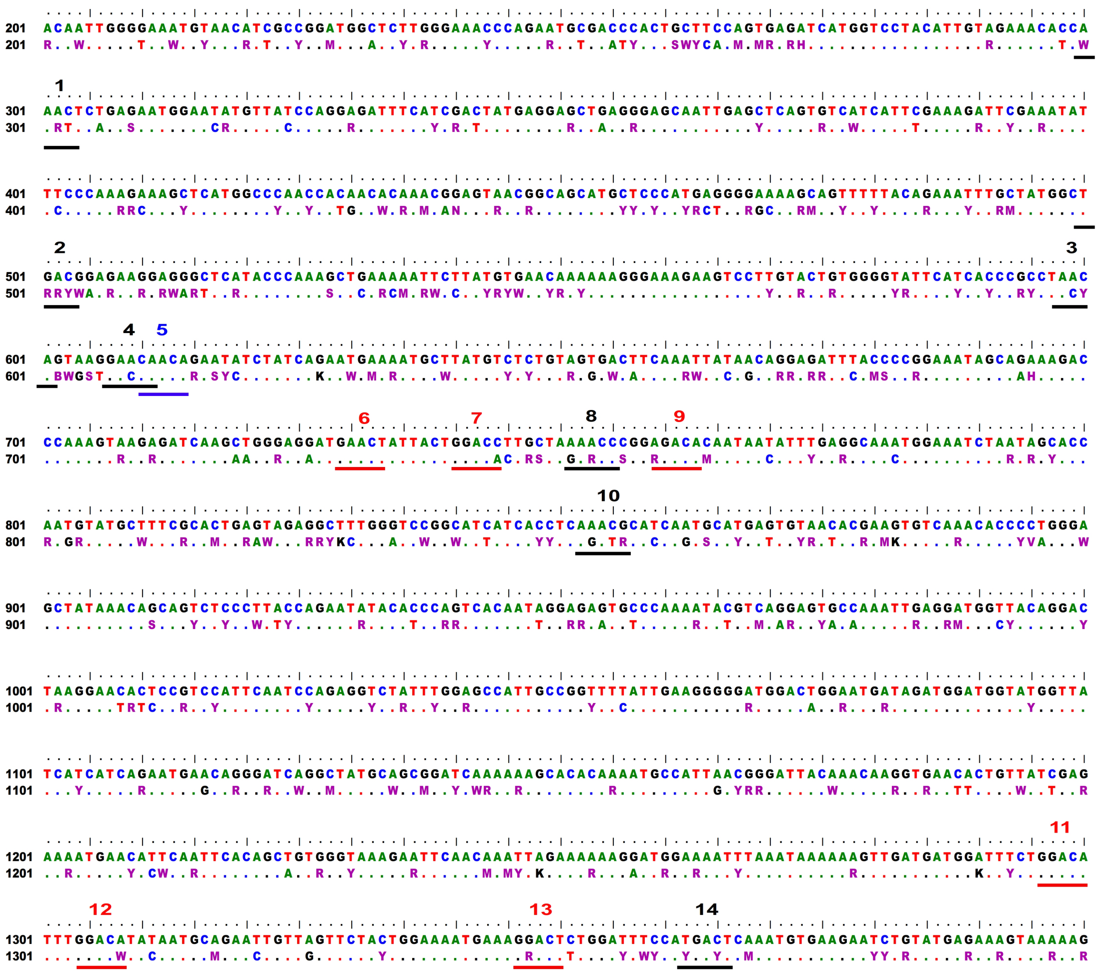

Supplementary Figure 1: DRACH motifs comparison between IAV-PR8 HA mRNA and the consensus sequence of the H1 subtype. Sequence alignment of the IAV-PR8 HA transcripts and consensus H1 with threshold frequency 85% are indicated. Nucleotide numbers are indicated on the left side. The alignment was generated using the Clustal W algorithm of the

MegAlign program (Lasergene, version 3.18). The conserved DRACH motifs indicted by red underlines and numbers, the non-conserved DRACH motifs indicated by black underlines and numbers, atypical 5th DRACH motif represented by blue underline. Identical residues are indicated by dots and sequence variation is denoted by a typical single-letter code or IUPAC degenerate nucleotide codes; R denotes A or G, Y denotes C or T, S denotes G or C, W denotes A or T, K denotes G or T, M denotes A or C, B denotes C or G or T, D denotes A or G or T, H denotes A or C or T, V denotes A or C or G.

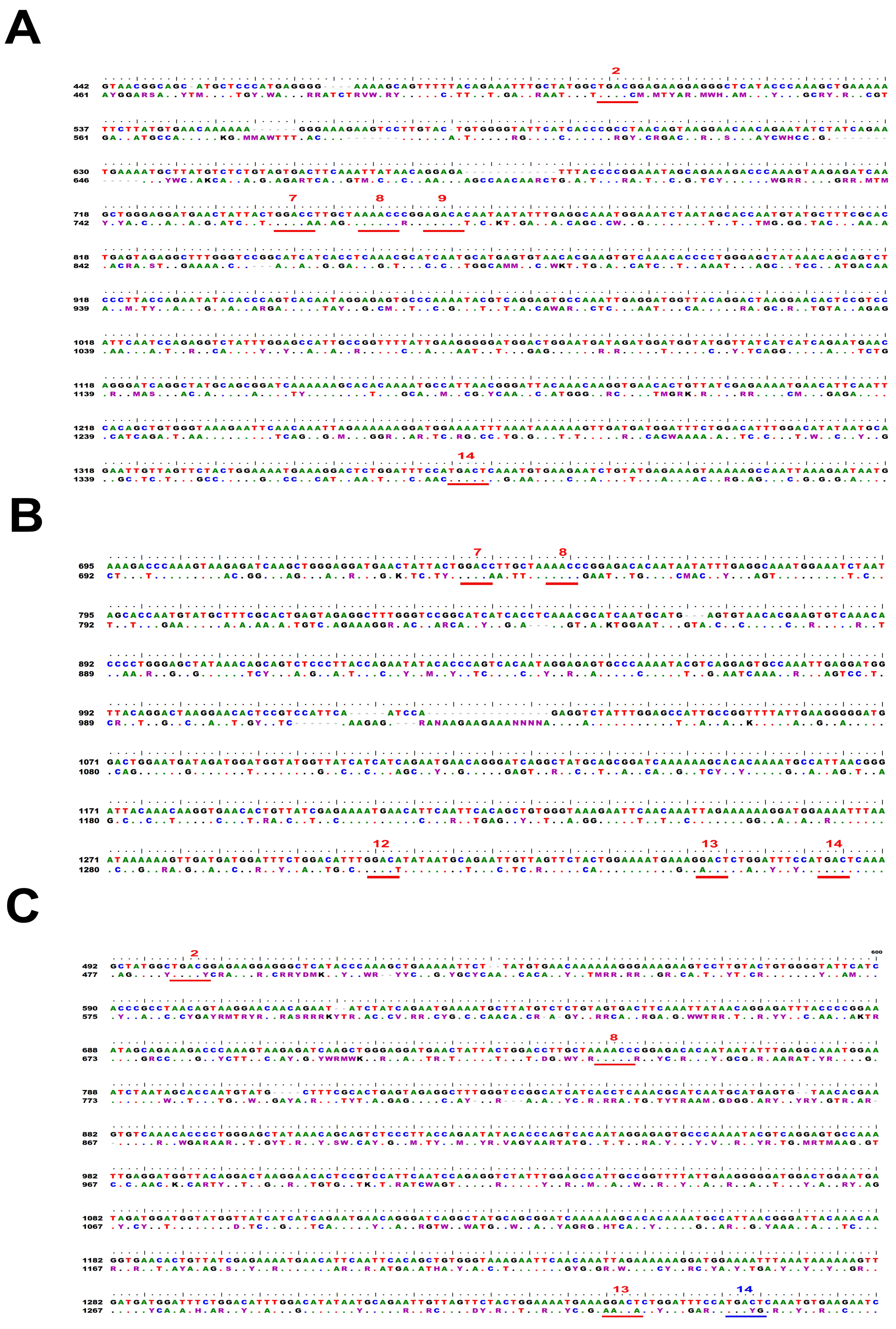

Supplementary Figure 2: DRACH motifs comparison between IAV-PR8 HA mRNA and the consensus sequences of various subtypes. Sequence alignment of the IAV-PR8 HA transcripts and consensus H3 (A), H5 (B), H9 (C) with threshold frequency 85, 75, and 90%, respectively, are indicated. Nucleotide numbers are indicated on the left side. The alignment

was generated using the Clustal W algorithm of the MegAlign program (Lasergene, version 3.18). The conserved DRACH motifs indicated by red underlines and numbers, atypical 14th DRACH motif represented by blue underline, and number are indicated (C). Identical residues are indicated by dots and sequence variation is denoted by a typical single-letter code or IUPAC degenerate nucleotide codes; R denotes A or G, Y denotes C or T, S denotes G or C, W denotes A or T, K denotes G or T, M denotes A or C, B denotes C or G or T, D denotes A or G or T, H denotes A or C or T, V denotes A or C or G.

Supplementary Table 1: Summary of conserved DRACHs among various species and viruses of H1 located on the vRNA.

vRNA	Seq no.	Motif1	Motif2	Motif3	Motif5	Motif6	Motif7	Motif8	Motif10	Motif11	Motif12
H1	25,576			✓95%		✓90%			✓95%		✓90%
Human H1N1	16,471			✓99%		✓99%			✓99%		✓99%
-Pandemic H1N1	10,993		✓50%	✓99%		✓99%			✓95%		✓99%
-Non-pandemic H1N1	5,490			✓99%		✓99%			✓99%		✓99%
Swine H1N1	4,366		✓75%	✓99%		✓85%			✓50%		✓95%
Human H1N2	43	✓95%		✓90%	✓90%	✓95%	✓95%	✓95%	✓99%	✓85%	✓99%
Swine H1N2	3,614	✓85%		✓95%		✓95%	✓75%	✓85%	✓95%	✓65%	✓95%
